# A Profile of Injuries Sustained by Firefighters: A Critical Review

**DOI:** 10.3390/ijerph16203931

**Published:** 2019-10-16

**Authors:** Robin Orr, Vinicius Simas, Elisa Canetti, Ben Schram

**Affiliations:** 1 Bond Institute of Health and Sport, Bond University, Gold Coast, QLD 4229, Australia; vsimas@bond.edu.au (V.S.); ecanetti@bond.edu.au (E.C.); bschram@bond.edu.au (B.S.); 2 Tactical Research Unit, Bond University, Gold Coast, QLD 4229, Australia

**Keywords:** fireman, firefighter, injury, tactical, occupational health

## Abstract

Firefighters, along with other tactical personnel, are at a high risk of work-related physical injury above that of the private sector. The aim of this critical narrative review was to identify, critically appraise and synthesise key findings from recent literature investigating firefighting musculoskeletal injuries to inform injury reduction programs. The methodological approach (search terms, databases, etc.) was registered with PROSPERO and reported following the Preferred Reporting Items for Systematic Reviews and Meta-Analyses (PRISMA) guidelines. Study quality was assessed using the Downs and Black checklist with scores graded according to the Kennelly grading system. Levels of evidence were ranked according to the Australian National Health and Medical Research Council. Of the 8231 studies identified, 17 met the criteria for inclusion. The methodological quality of the studies was ‘fair’ with a level of evidence of III-2. Reported injury rates ranged from 9% to 74% with the lower extremities and back the leading aggregated bodily sites of injury. Sprains and strains were the leading nature of musculoskeletal injury, often caused by slips, trips and falls, although muscle bending, lifting and squatting or muscle stressing were also prevalent. This review may inform injury reduction strategies and given that injuries reported in firefighters are similar to those of other tactical populations, safety processes to mitigate injuries may be of benefit across the tactical spectrum.

## 1. Introduction

Due to the unpredictable, varied and physical nature of firefighting duties, firefighters are at a high risk of work-related physical injury, suffering over three times the rates of those reported in the private sector [1]. Tasks performed by firefighters can include fire suppression, victim rescue, advancing a charged hose and climbing in and out of fire vehicles [2,3]. Often, tasks are performed within confined spaces, poor visibility and extreme heat [4], with temperatures reaching 50.9 °C at 0.3 m above the floor to 571.5 °C at ceiling height [2]. As a result of these occupational factors, firefighters can be at risk of not only fire-related injuries (i.e., burns) but musculoskeletal injuries as well [5]. Musculoskeletal injuries can include injuries to the shoulder, lower back, knee and ankle [5] and are similar to those suffered by other tactical occupations (i.e., military and law enforcement) [6,7]. In addition, and again like other tactical occupations, firefighters are required to carry external loads whilst performing key tasks [2].

The external loads carried by firefighters, being made up of personal protective clothing (PPE) and self-contained breathing apparatus (SCBA), can weigh between 17 and 25 kg [2,3]. This load is heavier than those carried by general duties police (approximately 10 kg) [8], similar to those carried by specialist police (22 kg) [9], but typically lighter than those carried by the military (up to 45 kg [10,11]). This occupational requirement to carry load is associated with causing injuries in tactical populations [12] and, as such, the loads carried by firefighters can increase their risk of physical injuries [13].

Injuries to firefighters incur costs to the individual and the firefighting organisation, in the form of resource, personnel and capability costs. One study [14], as an example, found that musculoskeletal injury, caused by a strain or sprain, could amount up to US $57,106 in medical costs. Furthermore, in terms of workforce loss, reduced work force strength and absenteeism (e.g., availability for shift work), firefighters were found to take twice as long to return to work following a musculoskeletal injury than workers in a private sector [1]. As such, reducing musculoskeletal injuries to firefighters can not only reduce fiscal costs but, just as importantly, maintain the integrity of the firefighter workforce. By profiling firefighter injuries by bodily sites, mechanisms and types of injuries sustained, informed prevention and rehabilitation practices can be developed. Therefore, the aim of this critical narrative review was to identify, critically appraise and synthesise key findings from recent literature investigating firefighting musculoskeletal injuries, in order to develop a profile of the injuries experienced by this unique population. Highlighting the key areas of injury is the first step in any evidence-based, injury minimisation strategy.

## 2. Methods

**Figure 1:**
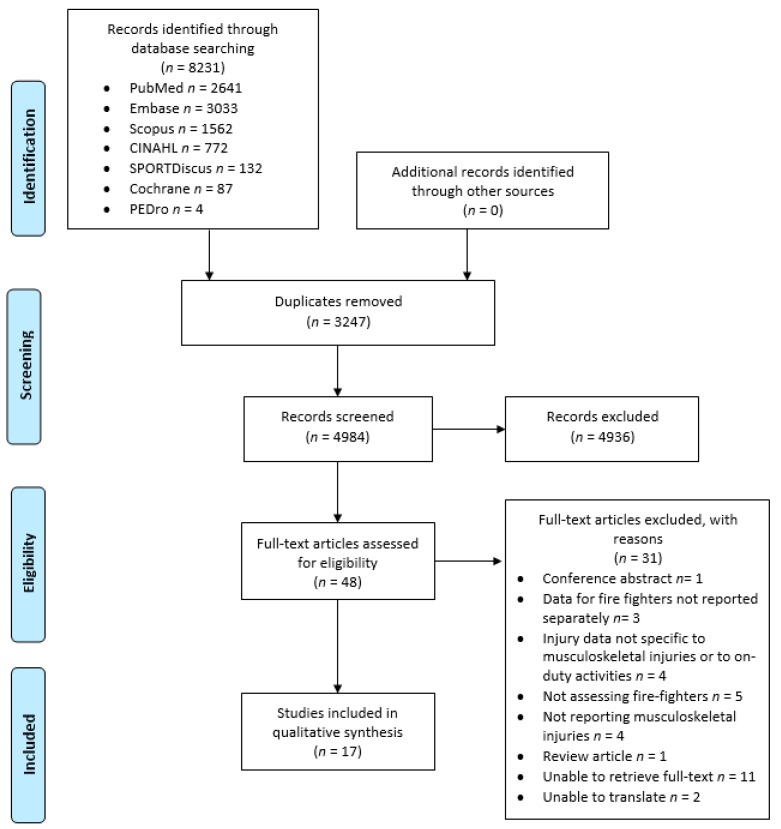
Preferred Reporting Items for Systematic Reviews and Meta-Analyses (PRISMA) flow diagram showing literature search, screening and eligibility results.

After collaboratively developing a list of key search terms and considered databases (Table 1), the methodological approach was registered with PROSPERO (#142258). Following review registration, the Preferred Reporting Items for Systematic Reviews and Meta-Analyses (PRISMA) guidelines (see Figure 1) were used to guide and report the database search and search outcomes [15]. To limit search bias, the search terms were kept broad to increase the inclusivity of studies.

To limit duplication bias and duplication of results, duplicate studies were removed during initial screening. Predefined inclusion and exclusion criteria (Table 2) were then used to screen the remaining studies by reviewing the study’s title and abstract. Where a determination could not be made using title and abstract, the full text was obtained and compared to the above criteria. Injury definitions, although varied, were described in six of the included studies [5,16,17,18,19,20] but were absent in the remaining studies. For the purpose of this review, all studies which focused on injuries sustained by firefighters whilst on duty were included.

The inclusion and exclusion criteria were established prior to screening commencing.

The Downs and Black checklist [21] was used to critically appraise included studies. This checklist is designed to assess the methodological quality of both randomised control trials and nonrandomised studies [21,22]. Using 27 questions, this checklist has been used to assess the methodological quality of studies in reviews involving tactical populations [6,21,23,24]. Question 27, which relates to statistical power, was modified from a scoring system of five down to zero points for the reporting of study power to a simplified one point for a ‘yes’ answer, indicating the authors had reported a sample size or power analysis, or zero points for a ‘no’ answer. As such, the maximum possible raw score for the Downs and Black checklist was reduced from a possible maximum of 32 points down to 28 points [21]. This approach, which has previously been used, was made due to the subjectivity of the Question 27 [21]. Several additional questions in the Down’s and Black checklist were removed from consideration (Questions 4, 8, 13, 14, 15, 19, 23 and 24) [21], given their focus on methods specific to experimental studies rather than the epidemiological, observational research designs of the included studies, and have again been previously reported in the literature [6]. This employment of these modifications to the Downs and Black checklist reduced the maximum possible total raw score to 20 points.

To grade the scores obtained using the Downs and Black checklist, the qualitative ratings of methodological quality proposed by Kennelly [25] were used. To account for the modifications made to the Downs and Black checklist, all scores, for both this study and those used to apply the grades proposed by Kennelly [25], were converted to percentages. Thus, the grading applied to this study was as follows: a score <45.4% related to ‘poor’ methodological quality; 45.4–61% a ‘fair’ methodological quality; and >61%, a ‘good’ methodological quality. The level of evidence for each study was graded using published Australian National Health and Medical Research Council (NHMRC) criteria [26]. These grades ranged from Level I (systematic review of all randomised control trials, highest level of evidence available) to Level IV (evidence obtained from case series, less reliable level of evidence) [26].

Following critical appraisal, key data, including participant demographics, injury definitions and main findings of relevance to the aims of this review were extracted and tabulated. From these key data, and in conjunction with further information provided in the original studies, a critical narrative synthesis was conducted. In the synthesis, the findings from each included study were considered in the light of the respective study’s methodological quality, represented by its Critical Appraisal Score (CAS), Kennelly [25] quality rating and NHMRC level of evidence.

## 3. Results

The database search results are reported in Table 3.

An overview of the results of the literature search, screening and selection processes is presented in the PRISMA flow diagram (Figure 1). An initial 8231 publications were identified in the search, with this number being reduced to 4984 studies following the removal of duplicates. Following further review of the studies by title and abstract, 49 full text publications were retrieved and evaluated in detail. Inclusion and exclusion criteria were then applied, with the final result being 17 publications considered to form the basis for this critical narrative review.

The final CAS detailing the methodological quality of each included study is listed in Table 4, along with the levels of evidence the studies provided and key study data (i.e., study aims and research design). Three studies were graded as being of ‘good’ methodological quality [19,27,28], twelve studies were graded as being of ‘fair’ methodological quality [5,14,16,17,18,20,29,30,31,32,33,34], and two studies were graded as being of ‘poor’ methodological quality [1,35]. Ranging from 20% [1] to 85% [19], the mean (± SD, standard deviation) CAS for methodological quality was 56.5% (±13.7%).

Common weaknesses were found across all included studies in the areas of reporting (three of eight questions), internal validity—bias (one of four questions) and internal validity—confounding/selection bias (two of five questions). As noted in Table 4, of the 17 studies included in this review, 12 [1,5,14,16,17,18,20,28,29,32,34,35] were determined to constitute level III-2 evidence, due to their use of a retrospective cohort study design. The remaining five studies [19,27,30,31,33] were cross-sectional and were therefore deemed to constitute level IV evidence.

Of the included studies, 10 were conducted in the United States [1,14,17,18,27,28,29,30,33,35], 2 in Canada [5,16], 1 in each of Greece [31], Poland [32], Australia [20], and the Republic of Korea [19], and 1 was an international study assessing individuals from United Kingdom, Ireland, North America, Australasia, and mainland Europe [33]. Apart from one study published in Greek [31], all studies were published in English. Table 5 provides details of each study’s injury definition, participants and main findings.

**Figure 2:**
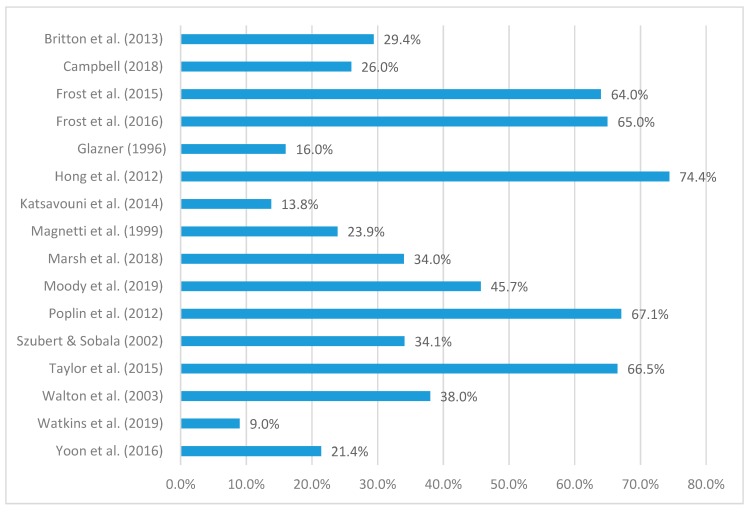
Musculoskeletal injury prevalence, by study.

There were varying ways that researchers gathered their injury data (Table 4). Three studies [14,29,34] collected data from workers’ compensation claims or reports, and the remaining studies [1,5,16,17,18,19,20,27,28,30,31,32,33,35] collected their data from injury databases, injury records filed by individual fire departments or surveys given to fire service personnel. The musculoskeletal injury prevalence was reported in 16 included studies [5,14,16,17,18,19,20,27,28,29,30,31,32,33,35] (Figure 2). The reported overall prevalence of musculoskeletal injury for fire fighters included in the studies reviewed varied from 9% [33] to 74.% [30], with no clear patterns evident based on particular population types or other contextual factors, for example, injury reporting processes. In only three studies [19,29,34], musculoskeletal injury was not the main type of injury reported. Only six [5,16,17,18,19,20] of the 17 included studies provided a clear definition of injury. Generally, the studies only reported the type of injury they were investigating, without providing a more clear and definitive description of exactly what comprised those injury types. This lack of clear injury definitions in most included studies prevented valid comparison of injury rates between studies and between the varying populations and contexts associated with the studies.

Five of the included studies [5,16,18,28,31] reported the body sites affected by recorded injuries. Of these, three studies [5,16,31] found that the most common site of injury was the back, with reported proportions of injuries ranging from 20% [16] to 32% [5]. The remaining two studies [18,28] reported the body site affected was the lower extremity, without specifying any particular region. Of the four studies [5,16,28,31] that examined how recorded injuries occurred, two [5,16] reported bending/lifting/squatting as the most common cause, and the other two [28,31] reported slips, trips and falls as the prevailing cause of recorded injuries. Four studies [5,16,17,18] reported the type of activity associated with injuries. Of these, two studies [5,16] reported general activities at the fire station as the main job activity associated with injuries, one [18] reported physical training activities as the main activity and one [17] firefighting. Two studies [5,14] investigated the association between musculoskeletal injuries and medical costs. One of the studies [5] reported musculoskeletal disorders as the main contributor to medical and compensation cost of injuries, responsible for 77% of the total amount. The authors of this study found the knee joint to be the costliest, followed by the back. In the other study [14], the mean workers’ compensation cost of strain/sprain injuries alone was 55% higher than the average for all causes.

## 4. Discussion

The aim of this review was to identify and critically appraise recently published studies investigating musculoskeletal injuries in firefighters and to synthesise and report their findings. Overall, the quality of the studies reviewed was considered of fair quality, with a mean CAS score of 56.5% ± 13.7%, with the majority of studies (71%) constituting level III-2 evidence. Whilst this score may seem low, it is typical of studies in this field which employ retrospective cohort or cross-sectional study designs [6]. The main findings of this critical review formed four main categories of results for discussion. These were: (1) musculoskeletal injury incidence in firefighter populations; (2) commonly injured body sites; (3) common natures of injury; and (4) common mechanisms of injury.

### 4.1. Musculoskeletal Injury Incidence in Firefighter Populaitons

The prevalence of firefighter musculoskeletal injuries identified in this review ranged from 9% [33] to 74.4% [30]. This wide range can be attributed to variations in, or lack of, injury definitions, the reporting mechanisms, the study duration and the means of data collection, all of which have the potential to influence reported rates [6,36]. Six [5,16,17,18,19,20] of the seventeen studies for example, provided a clear definition of injury, while studies collected their data from workers’ compensation claims or reports, injury databases, injury records filed by individual fire departments, or from surveys given to fire service personnel.

Two of the studies included in this review presented the injury rates as injuries per 1000 full-time employees per year [18,20]. Given the lack of information regarding injuries per period of time, this may underreport injuries, as longer durations would logically equate to an increased likelihood of injury reporting. In an Australian firefighter population, an average of 177 injuries per 1000 full-time employees per year were reported, with the data covering a nine-year period [20]. Likewise, rates of 177 (range of 136–215) injuries per 1000 employees per year have been reported in a population of US firefighters [18]. These rates are lower than those reported in a critical review of injuries sustained by law enforcement personnel by Lyons et al. [6], which ranged from 240 to 2500 per 1000 personnel per year across the 16 included studies. Within the Australian army, injury incidence rates in full-time personnel of 169.3 per 1000 personnel per year have been reported [37]. Although these injury incidence rates are lower than those reported in the firefighters in this review, underreporting rates of up to 49% have been noted in military personnel [38], and, as such, these military injury incidence rates may be conservative. Apart from the aforementioned potential factors that may have influenced the reporting rates, the varying natures of occupational tasks within different tactical fields (firefighter versus law enforcement versus military) may also contribute to observed differences in reported injury incidence rates.

### 4.2. Commonly Injured Body Site

The two most common aggregated sites of injury were the lower extremity and the back, with the lower extremity rates either being similar to [5,16,31] or exceeding [18,28] the back. However, of the three studies [5,16,31] that were more specific in injury site regions, the back presented as the leading site of injury, with reported proportions of injuries ranging from 20% [16] to 32% [5]. Of the lower limb injury sites, the proportion of knee and ankle [16] and knee and foot [31] injuries tended to be similar, although one study [5] reported knee injuries to be approximately twice as high as ankle injuries (22.6% and 10.7%, respectively). Shoulder injuries represented the lowest proportion of injuries by bodily site in two [16,31] of the three studies reporting shoulder injuries but were greater than those of ankle injuries in a third study [5] (ankle = 10.7%; shoulder = 14.5%). When considering the sites of injury, the findings of Frost et al. [5], who identified the knee joint to be the most costly followed by the back, warrant consideration.

The findings of this study differ from those in law enforcement populations but are similar to those in military populations. In law enforcement, the upper extremity was found to be the most common injury site, representing 33–43% of reported musculoskeletal injuries [6]. Conversely, and similar to the findings this study, rates in the military have reported the lower extremity and back to be the most common sites of injury [12,39,40,41,42,43]. A potential reason for the differences in bodily sites between these tactical populations would not only be differences in occupational tasks, but also occupational load carriage requirements as law enforcement generally wears lighter loads over longer periods, while firefighters and military wear heavier loads intermittently.

### 4.3. Common Nature of Injury

This review found the most common nature of injury to be reported were sprains and strains [17,18,20,27,28,29,30,31,32,35], ranging from 16% [29] up to 74% [30]. While two studies did find sprains and strains to be the second most common nature of injury, with the numbers of these injuries being preceded by wounds, cuts and bleeding (42.3%) [19] or lacerations and contusions (28.9%) [34], sprains and strains still presented as the leading nature of musculoskeletal injury. In quantifying the costs of these types of injuries, Walton et al. [14] found a mean cost associated with sprains and strains to total $8031 per person. As such, when considering that 74% of reported injuries may be of this nature, the costs associated with these injuries can be substantial.

These findings are similar to those reported in law enforcement with sprains and strains likewise being the leading nature of injuries [6]. Of note, however, the rates of injuries reported in law enforcement were generally greater, ranging from 42.4% [44] to 94.6% [45]. Similarly, sprains and strains are typically the most common nature of injury within military populations [38,46]. As such, the results of this study suggest that, like law enforcement and military personnel, the nature of firefighter musculoskeletal injuries is most commonly sprains and strains. As such, means of mitigating, or reducing the severity of, or optimising the rehabilitation for, these types of injuries may serve to aid multiple tactical organisations.

### 4.4. Common Mechanisms of Injury

Of the five studies [5,16,20,28,31] that examined how recorded injuries occurred, slips, trips and falls were noted as being the most common mechanism of injury in two studies [28,31]. In two studies [5,16], slips, trips and falls were the second most common mechanism following bending, lifting and squatting, with differences between these two mechanisms of little margin. For example, Frost et al. [16] found 23% of injuries were caused by bending, lifting and squatting, while 21% of injuries were caused by slips, trips and falls. In the fifth study, muscular stress made up three of the top five mechanisms of injury, while falls (i.e., fall on the same level and fall from a height) made up the remaining two of the top five mechanisms [20]. When these mechanisms were collapsed by commonality, muscle stress accounted for 74 injuries per 1000 full time employment firefighters per year and slips, trips and falls 40 injuries. Of note, Britton et al. [28] found that slips, trips and falls were the mechanism of injury accountable for almost half of all sprains and strains (49%) and fractures and dislocations (43%). Where the compensation costs associated with these mechanisms of injury were considered, they were reported to average US $8662 per person [14]. It should be noted that the studies whereby slips, trips and falls were the most common site of injuries were of wildland firefighters [28] or firefighters from across Greece [31], which may have included wildland firefighters. The remaining three studies [20,28,31], where other mechanisms (like bending, lifting and squatting or muscle stressing) preceded slips, trips and falls were of urban firefighters.

The mechanisms of injury identified in this review are similar to those reported in military populations [46,47,48]. For example, in the Australian Defence Force, slips, trips and falls were identified as the leading mechanism of injury (21.4%) [46]. Considering this, body stressing, which was the fourth most common mechanism of injury (16.3%), was the mechanism associated with the highest number of working days lost (26.9%), followed by slips, trips and falls (25.2%). Even in an operational military combat theatre, falls have been identified as the leading site of nonbattle injuries, accounting for 21.3% of injuries sustained by US service members deployed to Iraq and Afghanistan over a 12-year period [48]. As such, strategies to prevent slips, trips and falls, as well as muscle stressing, may impact both firefighter and military populations.

Of the four studies [5,16,17,18] that reported the type of activity associated with injuries, two studies by the same authors [5,16] reported general activities at the fire station as the main job activity associated with injuries, one [18] reported physical training activities (33%) as the leading cause and one [17] listed firefighting as being the leading cause (38%). One reason for these differences in reported mechanisms comes from the site of data collection. For example, in the study by Marsh et al. [17], the data were of firefighters treated in emergency departments. Conversely, the data which informed the study by Poplin et al. [18] came from a fire department’s injury reports. Furthermore, when comparing these studies, only the two studies by Frost et al. [5,16] use the same criteria to report the nature of the duties. Where Poplin et al. [18] used categories which included patient transport and training and drilling, Marsh et al. [17] used the categories firefighting, patient care and training, and Frost et al. [5,16] used job duties categories like fire station, training centre and gymnasium. The lack of mechanism standardisation across studies is evident and presents as a challenge in identifying and therefore applying mitigation strategies that may transcend beyond an individual department.

### 4.5. Limitations and Future Research

Two limitations are identified in this review: Firstly, the nature of the studies employed to investigate injuries in firefighters was typically retrospective in design, and they were either cross-sectional or cohort studies. This combination of design features meant that there was an increased risk of bias [49,50] that may be associated with the individual study’s findings. Secondly, the variables across studies often differed, from injury definition (if present), to sample duration, to the bodily sites, natures and mechanisms of injury categories used by the studies. This variability makes establishing a detailed volume of evidence difficult without the use of broad categories (like ‘lower extremity’ as a bodily site), which reduces result sensitivity. While these challenges are not new and have been reported in the literature [6,51], they do highlight the need for future research to be conducted with better methodological quality. Larger, prospective cohort studies using consistent injury definitions so as to allow for results that can be applied across the firefighter population serve as an example. In addition, providing injury rates per unit of time and using comprehensive injury reporting protocols and databases would enable a more robust body of literature in this area. Notwithstanding these limitations, this review does take the crucial first step in identifying these differences so as to inform future planning.

## 5. Conclusions

Similar to military personnel, the lower extremities and back were the leading bodily sites of injury, with the knee in general being the most common site of injury to the lower limbs. Like in other tactical populations, sprains and strains were a leading nature of musculoskeletal injury. Often the more common causes of injury were slips, trips and falls; however, muscle bending, lifting and squatting or muscle stressing were also prevalent, with the findings of this review leading to a suggestion that the causes could vary depending on firefighter duties (e.g., wildland or structural). The types of activity being undertaken at the time of injury were varied due to lack of consistent terms being used and were identified as being due to general activities at the fire station, physical training activities or firefighting itself. There was considerable variability in injury definition and categorisation, which impacts the ability to build a volume of evidence regarding firefighter injuries and highlights the need for future research to be conducted with better methodological quality. However, there do appear to be some similarities between the injuries presenting in firefighters and those of other tactical populations, and as such, means of mitigating and rehabilitating these injuries may be of benefit across the tactical spectrum. The similar injuries and risk factors across tactical environments should therefore be addressed collectively for mutual benefit.

## Figures and Tables

**Table 1:** Details of literature search: databases used and search terms (completed on 08 July 2019).

Database	Search Terms
PUBMED	(firefighter OR firefighters OR “fire fighter” OR “fire fighters” OR “Firefighters” [Mesh] OR “first responder” OR “first responders” OR “emergency responder” OR “emergency responders” OR “Emergency Responders” [Mesh] OR “fire and rescue” OR “smoke jumpers” OR fireman OR firemen) AND (injury OR injuries OR “Wounds and Injuries” [Mesh])
CINAHL	(firefighter OR firefighters OR “fire fighter” OR “fire fighters” OR (MM “Firefighters+”) OR “first responder” OR “first responders” OR “emergency responder” OR “emergency responders” OR “fire and rescue” OR “smoke jumpers” OR fireman OR firemen) AND (injury OR injuries OR (MM “Wounds and Injuries+”))
EMBASE	(‘firefighter’/exp OR firefighter OR firefighters OR ‘fire fighter’/exp OR ‘fire fighter’ OR ‘fire fighters’/exp OR ‘fire fighters’ OR ‘firefighters’/exp OR ‘firefighters’ OR ‘first responder’ OR ‘first responders’ OR ‘emergency responder’/exp OR ‘emergency responder’ OR ‘emergency responders’/exp OR ‘emergency responders’ OR ‘fire and rescue’ OR ‘smoke jumpers’ OR ‘fireman’/exp OR fireman OR firemen) AND (‘injury’/exp OR injury OR ‘injuries’/exp OR injuries OR ‘wounds and injuries’/exp)
COCHRANE	(firefighter OR firefighters OR “fire fighter” OR “fire fighters” OR [mh Firefighters] OR “first responder” OR “first responders” OR “emergency responder” OR “emergency responders” OR [mh “Emergency Responders”] OR “fire and rescue” OR “smoke jumpers” OR fireman OR firemen) AND (injury OR injuries OR [mh “Wounds and Injuries”])
SCOPUS	( TITLE-ABS-KEY(“firefighter”) OR TITLE-ABS-KEY(“firefighters”) OR TITLE-ABS-KEY(“fire fighter”) OR TITLE-ABS-KEY(“fire fighters”) OR INDEXTERMS(“Firefighters”) OR TITLE-ABS-KEY(“first responder”) OR TITLE-ABS-KEY(“first responders”) OR TITLE-ABS-KEY(“emergency responder”) OR TITLE-ABS-KEY(“emergency responders”) OR INDEXTERMS(“Emergency Responders”) OR TITLE-ABS-KEY(“fire and rescue”) OR TITLE-ABS-KEY(“smoke jumpers”) OR TITLE-ABS-KEY(“fireman”) OR TITLE-ABS-KEY(“firemen”) ) AND ( TITLE-ABS-KEY(“injury”) OR TITLE-ABS-KEY(“injuries”) OR INDEXTERMS(“Wounds and Injuries”) )
SPORTDISCUS	(firefighter OR firefighters OR “fire fighter” OR “fire fighters” OR “first responder” OR “first responders” OR “emergency responder” OR “emergency responders” OR OR “fire and rescue” OR “smoke jumpers” OR fireman OR firemen) AND (injury OR injuries OR DE “WOUNDS & injuries”)
PEDRO	“firefighter*” AND “injur*”

**Table 2:** Inclusion and exclusion criteria and examples of excluded studies.

**Inclusion Criteria**	**Example/s**
Study was focused on firefighters	Studies involving wildland firefighters, urban firefighters, career firefighters, volunteer firefighters, fire service personnel
Study examined musculoskeletal injuries occurring to, or in, a firefighter population while on duty	Studies examining musculoskeletal injuries, musculoskeletal disability, injury epidemiology, injury rates, injury incidence
**Exclusion Criteria**	**Example/s**
Study involved participants who were not firefighters	Studies involving emergency medical responders, first responders, or other population, without data specific for firefighters
Study included only injuries that were not musculoskeletal injuries while on duty	Studies which only examined occupational injuries without data for musculoskeletal injuries, or studies which examined, recreational injuries, sporting injuries, fatalities, chemical hazards, mortality, homicide, suicide, mental illness
Review article, or study reported as an abstract only	

**Table 3:** Databases and search results prior to screening and removal of duplicates.

Database	Identified Studies (*n*)
PUBMED	2641
CINAHL	772
EMBASE	3033
COCHRANE	87
SCOPUS	1562
SPORTDISCUS	132
PEDRO	4

**Table 4:** Key information regarding the design, aims, data sources, critical appraisal score and levels of evidence of each included study.

Authors (Year) and [Reference]	Title	Aim/Objective/Hypothesis	Study Design	Data Collection Method	Downs and Black Score	Level of Evidence *
Britton et al. (2013) [28]	Epidemiology of injuries to wildland firefighters	Examine nonfatal wildland firefighter injuries reported to the US Department of Interior (DOI) from 2003 to 2007.	Retrospective Cohort	Database	70%	III-2
Campbell (2018) [35]	US Firefighter Injuries on the Fireground, 2010–2014	Profile US firefighter injuries occurring on the fireground from 2010 to 2014.	Retrospective Cohort	Database	45%	III-2
Frost et al. (2015) [16]	Firefighter injuries are not just a fireground problem	Characterise the injuries sustained by members of a large Canadian metropolitan fire department over a 5-year span (2007–2011)	Retrospective Cohort	Database	50%	III-2
Frost et al. (2016) [5]	The cost and distribution on firefighter injuries in a large Canadian Fire Department	Categorise the cost of injuries filed in 2012 by firefighters from a large department by job duty, injury type, body part affected, and the general motion pattern employed at the time of injury.	Retrospective Cohort	Database	60%	III-2
Glazner (1996) [29]	Factors related to injury of shiftwork fire fighters in the Northeastern United States	Identify factors involved in injuries sustained by fire fighters in three different municipal fire departments.	Retrospective Cohort	Database	60%	III-2
Hong et al. (2012) [30]	Occupational Injuries, Duty Status, and Factors Associated With Injuries Among Firefighters	Assess the type of occupational injuries, duty status, and factors associated with injuries among firefighters.	Cross-sectional	Self-report/Internet-based survey	60%	IV
Katsavouni et al. (2014) [31]	The type and causes of injuries in firefighters	Investigate the nature and causes of occupational injuries in firefighters.	Cross-sectional	Self-report	55%	IV
Magnetti et al. (1999) [34]	Injuries to Volunteer Fire Fighters in West Virginia	Describe the distribution of occupational injuries to volunteer fire fighters (VFFs) by demographic characteristics, presenting history, time, and geographic location, using the West Virginia State Worker’s Compensation database.	Retrospective Cohort	Database	50%	III-2
Marsh et al. (2018) [17]	Nonfatal Injuries to Firefighters Treated in U.S. Emergency Departments, 2003–2014	Enhance current knowledge by providing national estimates of nonfatal injuries to firefighters treated in US emergency departments	Retrospective Cohort	Database	55%	III-2
Moody et al. (2019) [27]	Descriptive analysis of injuries and illnesses self-reported by wildland firefighters	Understand types of injuries and illnesses wildland firefighters (WLFFs) sustain during the fire season.	Cross-sectional	Web-based self-reported questionnaire	75%	IV
Poplin et al. (2012) [18]	Beyond the fireground: injuries in the fire service	Explore injuries not only on the fireground but also during other fire service activities.	Retrospective Cohort	Database	55%	III-2
Seabury and McLaren (2012) [1]	The Frequency, Severity, and Economic Consequences of Musculoskeletal Injuries to Firefighters in California	# Describe the average frequency and severity of work-related musculoskeletal disorders (MSDs) experienced by firefighters in California. # Study the impact of work-related MSDs on the earnings and employment of firefighters several years after injury. # Evaluate the impact of reforms to the disability rating system on the ratings of firefighters with permanently disabling MSDs. # Assess whether reforms to the medical delivery system affected the employment outcomes of firefighters with MSDs.	Retrospective Cohort	Database	20%	III-2
Szubert and Sobala (2002) [32]	Work-related injuries among firefighters: sites and circumstances	Determine the injury ratio, causes and duration of temporal work disability from on-duty injuries among firefighters, taking into account the site and circumstances of their occurrence.	Retrospective Cohort	Database	55%	III-2
Taylor et al. (2015) [20]	A Retrospective Evaluation of Injuries to Australian Urban Firefighters (2003 to 2012): Injury Types, Locations, and Causal Mechanisms	Evaluate injury trends within Australian firefighters.	Retrospective Cohort	Database	50%	III-2
Walton et al. (2003) [14]	Cause, Type, and Workers’ Compensation Costs of Injury to Fire Fighters	A better understanding of the costs of injury to firefighters, and how those costs relate to the cause and the nature of those injuries can help inform policy decisions regarding the occupational health of firefighters (Musich et.al., 2001). Such information can serve to document financial incentives to infuse dollars into firefighter injury prevention and suggest priority areas for further study.	Retrospective Cohort	Database	55%	III-2
Watkins et al. (2019) [33]	Women Firefighters’ Health and Well-Being: An International Survey	Identify specific health and well-being issues that women firefighters may experience as part of their daily working practices. Issues identified from this under-represented population can drive future research, education, and strategy to guide safety and health practices.	Cross-sectiona	Self-report	60%	IV
Yoon et al. (2016) [19]	Characteristics of Workplace Injuries among Nineteen Thousand Korean Firefighters	Provide as comprehensive an evaluation as possible to aid in improving safety strategies for firefighters, as well as to improve their health and well-being.	Cross-sectional	Self-report	85%	IV

* National Health and Medical Research Council (NHMRC) guidelines used to determine the level of evidence [26].

**Table 5:** Injury definitions, participants and main findings from each included study.

Authors [Reference]	Year	Title	Injury Definition	Participant Details	Main Findings
Britton et al. [28]	2013	Epidemiology of injuries to wildland firefighters	No Injury definition	# Over 200,000 wildland firefighters (USA) in the 5-year period from 2003 to 2007 # Age at injury = 17 to 65 years # Total occupational injuries = 1301	# Slips/trips/falls were the mechanism for almost half of all sprains and strains (49%) and fractures and dislocations (43%). # Of all injuries: - Fractures/dislocations = 51 (3.9%). - Sprains/strains = 382 (29.4%, most common type). # Almost two-thirds of all injuries (65%) occurred during the peak season. # For all injuries, the lower extremity was the most common body part involved (35%). # Back injuries represented slightly less than 10% of all injuries reported but comprised 21% of all injuries caused by equipment/tools/machinery. # Of the 121 back injuries reported, 29 (16%) were considered severe (data not shown).
Campbell [35]	2018	US Firefighter Injuries on the Fireground, 2010-2014	No Injury definition	# 1,121,630 Firefighters from USA (2010–2014) - Carrer = 345,180 - Volunteers = 776,450 - Career males = 334,050 - Career females = 20,590 - Volunteer males = 717,800 - Volunteer females = 70,450 # Total 30,290 injuries/year	# Leading cause of injuries = overexertion or strain (26%) # Strain or sprain was the leading primary symptom (28%)
Frost et al. [16]	2015	Firefighter injuries are not just a fireground problem	A reportable injury was defined in accordance with the Occupational Health and Safety regulations for Alberta, namely medical treatment, restricted work duties or lost time.	# Calgary Fire Department (western Canada) #1311 injuries (2007–2011) # In 2011:- 1363 personnel - 37 stations - Age = 38 (SD 9) years - Height = 1.80 (SD 0.06) m - Body mass = 89 (SD 11) kg - 2% were women - 100,695 responses to 50,520 incidences - 204 injuries	# Musculoskeletal disorders (MSD) = 845 (64% of all injuries, 2007-2011): - Ankle = 9.5% - Back = 20.1% - Knee = 10.6% - Shoulder = 8% - Other (neck, hip, elbow, wrist, hand, foot, abdomen, chest, arm, leg, and groin) = 15.3% # Job site/occupation: - Fire station = 37.9% - Physical training = 26.6% # Mechanism: - Bending/Lifting/Squatting = 23.2% - Slipping/Tripping/Falling = 21.3%
Frost et al. [5]	2016	The cost and distribution on firefighter injuries in a large Canadian Fire Department	A reportable injury was defined in accordance with the Occupational Health and Safety regulations for Alberta, namely medical treatment, restricted work duties or lost time, which was defined as missing one or more shifts because of an occurrence.	# Calgary Fire Department (western Canada) # 1289 personnel# 38 stations # 102,632 responses to 52,918 incidences # 244 injuries	# Musculoskeletal disorders (MSD) = 159 (65% of all injuries): - Ankle = 10.7% - Back = 32.1% - Knee = 22.6% - Shoulder = 14.5% - Other (neck, hip, elbow, wrist, hand, foot, abdomen, chest, arm, leg, and groin) = 20.1 # Job site/occupation: - Fire station = 31% - Physical training = 28%# Mechanism: - Bending/Lifting/Squatting = 23% - Slipping/Tripping/Falling = 18% # MSD = 77% of medical and compesation costs: - 28% = knee - 18% = back
Glazner [29]	1996	Factors related to injury of shiftwork fire fighters in the Northeastern United States	No Injury definition	# 447 career fire fighters of 3 fire departments (USA) # 171 injuries	# Sprains, strains, or pain = 16% # Back, neck, or knee injuries = 4% # Fractures = 4%
Hong et al. [30]	2012	Occupational Injuries, Duty Status, and Factors Associated With Injuries Among Firefighters	No Injury definition	# 437 fire fighters from 34 fire departments (USA), from 2010 to 2011 # 92.4% male # 80% Caucasian # Mean age = 44.9 (SD 8.1) years # Years worked in fire services = 17.4 (SD 8.2) years # Occupational accidents/injuries n = 285	# Muscle strains/sprains = 212 (74.4%) # Back injury = 153 # Broken bones = 35
Katsavouni et al. [31]	2014	The type and causes of injuries in firefighters	No Injury definition	# 3289 full-time firefighters (Greece) # Age = 24 to 60 years (mean 36.4, SD 6.19) # 96.3% males # 502 individuals reported injury at work	# Lumbar injuries = 107 # Muscle strains = 69 (13.8%) # Foot injuries = 41 # Knee injuries = 41 # Leg injuries = 28 # Shoulder injuries = 19 # Chest-rib injuries = 14 # Neck injuries = 11 # Main mechanism = slip/trip/fall
Magnetti et al. [34]	1999	Injuries to Volunteer Fire Fighters in West Virginia	No Injury definition	# Volunteer fire fighters (VFFs) of West Virginia (USA) # Age = 27.6 (SD 9.96) years # 343 workers’ compensation claims for occupational injuries for the fiscal year 1992 # Injury rate = 36.07 per 1000 responses	# Lacerations and contusions = 28.9% # Strains and sprains = 23.9%
Marsh et al. [17]	2018	Nonfatal Injuries to Firefighters Treated in U.S. Emergency Departments, 2003-2014	The term “injury” was used to refer to injuries, illnesses, and exposures	# USA # 95% male # 35% aged between 30-39 years # 351,800 injuries (2003–2014)	# Sprain/Strain = 34% # Fracture/dislocation = 5% # Training and patient care activities both most often resulted in sprains and strains
Moody et al. [27]	2019	Descriptive analysis of injuries and illnesses self-reported by wildland firefighters	No Injury definition	# 284 wildland firefighters (WLFFs) (USA) # Of 254 WLFFs who reported at least one injury: - 87.4% Male - 38.1% aged 35–44 years # 453 injuries and illnesses (over 5 years)	# Joint sprain = 25.4% # Muscle strain = 15.2% # Fracture/dislocation = 7.1% # Tendinitis = 5.1% # Muscle cramp, spasm = 1.8%
Poplin et al. [18]	2007	Beyond the fireground: injuries in the fire service	A reportable injury is defined in accordance with Occupational Safety and Health Administration (OSHA) regulations (29 CFR, 1904.7) (i.e., medical treatment, restricted work time, or lost work time), in addition to any injury that occurred to specific body regions. The surveillance database includes internally documented injuries (i.e., those deemed non-OSHA reportable, but recorded in the Tucson Fire Department system), which had no immediate loss of job function or capabilities, but are documented in the event the injury later progresses to a point requiring a report and or treatment (e.g., due to cumulative or repeated trauma)	# Approximately 650 fire service personnel (USA), including firefighters, paramedics, engineers, inspectors, battalion chiefs # Average of 41 years of age # 5% were women # 76.4% non-Hispanic white # 902 injuries in 409 individuals (2004–2009) # Annual incidence rates ranging between 13.6 and 21.5 injuries per 200,000 h (equivalent to 100 full-time employees) # Mean age of those injured = 37.9 years (20–64 y)	# Sprain, strain = 605 (67.1%) - 44.6% = lower extremity- 32.2% = back/spine - 65.3% = no lost time - 100% = minor - 25.6 % = firefighters - 22.2% = paramedics - 41.8% during physical exercise # Fireground operations = 92 injuries - 40.2% = sprain/strain - 2.2% = fracture/dislocation
Seabury and McLaren [1]	2012	The Frequency, Severity, and Economic Consequences of Musculoskeletal Injuries to Firefighters in California	No Injury definition	# Firefighters from California	# Firefighters are 3.5 times more likely to suffer a workplace injury and 3.8 times more likely to suffer a work-related MSD than a private-sector worker. # Firefighters take 1.4 times longer to return to work than workers in the private sector for all injuries; this difference skyrockets for MSDs, as firefighters take twice as long to return to work. # The median number of days away from work after an MSD is 1.8 times greater for an MSD than for any other injury for firefighters, whereas this ratio is only 1.25 for private-sector workers. # Both the frequency and the severity of injuries, particularly MSDs, are worse for older firefighters than for younger firefighters. # Older firefighters are 10.4 times more likely to suffer an MSD than are private-sector workers, and they take more than four times longer to return to work.
Szubert and Sobala [32]	2002	Work-related injuries among firefighters: sites and circumstances	No Injury definition	# 1503 firefighters from 29 fire stations in Poland # In 1994: - Mean age = 32 (SD 6.5) years # 352 injuries (1994–1997)	# 25% were on-duty: - 10.2% = Fractures - 6.8% = Fractures of lower limb - 34.1% = Dislocation, sprains, and strains
Taylor et al. [20]	2015	A Retrospective Evaluation of Injuries to Australian Urban Firefighters (2003 to 2012): Injury Types, Locations, and Causal Mechanisms	Work-related injuries were defined as physical and psychological (mental health) conditions that arose during, or as a consequence of, employment as a firefighter.	# 6998 Australian firefighters # In 2012: - 95.9% males # 1,225,218 callouts, 6997 injuries (2003 to 2012)	# Joint and muscle sprains and strains = 66.5%: - 177 cases per annum per 1000 full-time firefighters (FTE)
Walton et al. [14]	2003	Cause, Type, and Workers’ Compensation Costs of Injury to Fire Fighters	Injury was defined as any mild physical harm (e.g., bruises), or any major physical harm involving outpatient or inpatient treatment	# 13,680 firefighters (USA) # Average age = 35 years # 96% male # 1343 injuries (1992 to 1999)	# Strains and sprains account for 38% of the injuries claimed by firefighters # 83% of injuries with a cause of overexertion have a nature of injury of strain or sprain # Mean costs related to strain/sprain: - Medical = $3023 - Total = $8031
Watkins et al. [33]	2019	Women Firefighters’ Health and Well-Being: An International Survey	No injury definition	# 840 women firefighters from 14 countries (UK, Ireland, North America, Australasia, mainland Europe) # Age = 40 years (SD 9) # Time as firefighter = 13 years (SD 8)	# Musculoskeletal injuries, including work-related upper and lower limb and back injuries, were reported by 9–23% of women firefighters.
Yoon et al. [19]	2016	Characteristics of Workplace Injuries among Nineteen Thousand Korean Firefighters	The occurrence of workplace injuries was defined when the injuries required hospital care: if the injuries did not require hospital care, they were not counted. These criteria were applied to all types of injuries and events including car accidents.	# 19,119 Korean firefighters # Age 20 to 59 years # 2230 injured firefighters	# Most prevalent = wound, cut, bleeding, bruise (*n* = 1728; 42.3% of all injuries) # Fracture n = 368 (9% of all injuries) # Strain, sprain, muscular pain n = 876 (21.4% of all injuries)

SD = standard deviation; MSD = musculoskeletal disorder; VFF = volunteer firefighter.
